# Preemptively planned en bloc resection of an extensive right adrenal pheochromocytoma involving the right hepatic division, caval thrombus and segmental caudal vena cava in a dog with Budd−Chiari‐like syndrome

**DOI:** 10.1002/vms3.1110

**Published:** 2023-03-13

**Authors:** Ryo Takeuchi, Kumiko Ishigaki, Orie Yoshida, Naoki Sakurai, Kazuyuki Terai, Tatsuya Heishima, Kazushi Asano

**Affiliations:** ^1^ Laboratory of Veterinary Surgery, Department of Veterinary Medicine, College of Bioresource Sciences Nihon University Fujisawa Japan

**Keywords:** adrenal tumour, Budd−Chiari‐like syndrome, dog, pheochromocytoma, surgery

## Abstract

**Background:**

Surgical resection is the treatment of choice for canine adrenal pheochromocytomas (PHEOs). Information on en bloc resection of adrenal PHEO with tumour thrombus, right hepatic division and segmental caudal vena cava (CVC) running through the adrenal tumour and right hepatic division is limited.

**Objective:**

To describe the preemptively planned en bloc resection of an extensive right adrenal PHEO involving the right hepatic division, the caval thrombus and the segmental CVC in a dog with Budd−Chiari‐like syndrome (BCLS).

**Methods:**

A 13‐year‐old castrated male miniature dachshund was referred for surgical treatment due to anorexia, lethargy and severe abdominal distension caused by abundant ascites. Preoperative computed tomography (CT) revealed a large mass in the right adrenal gland with a large caval thrombus obstructing the CVC and hepatic veins, which caused BCLS. Additionally, collateral vessels were formed between the CVC and azygos veins. No findings suggested obvious metastases. Based on CT findings, an en bloc resection of the adrenal tumour with caval thrombus, right hepatic division and segmental CVC was planned.

**Results:**

The preoperatively planned resection was feasible; the tumour was completely resected grossly. The operation time and total Pringle manoeuvre time were 162 min and 16 min 56 s, respectively. There was no postoperative hindlimb oedema, renal dysfunction, ascites or abdominal distention. The patient's clinical signs, including appetite, fully improved. Hospitalization lasted 16 days. However, the patient died on the 130th postoperative day due to suspected metastases and cachexia.

**Conclusions:**

Even in case of an extensive infiltration of adrenal PHEO causing BCLS, an en bloc resection might be successfully achieved based on the preoperative CT findings speculating the collateral vessels formed for caudal venous return.

## Graphical abstract

1. An extensive caval thrombus associated with adrenal PHEO may cause Budd‐Chiari‐like syndrome (BCLS).
2. Information on en bloc resection of adrenal PHEO including tumor thrombus, segmental liver, and caudal vena cava, is limited.
3. In case of adrenal PHEO with BCLS, the en bloc resection is suggested to be feasible based on the preoperative CT findings speculating the collateral vessels formed for caudal venous return.

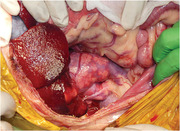

## 1 INTRODUCTION

In dogs, malignant adrenal tumours, including pheochromocytoma (PHEO) and adrenocortical adenocarcinoma, may invade the surrounding veins. Approximately 10−48% (Massari et al., 2011; Lang et al., 2011; Pey et al., 2022; Schwartz et al.; 2008; Schultz et al., 2009; Barthez et al., 1997; Yoshida et al., 2016; Nagumo et al., 2020; Davis et al., 2012) of canine adrenal tumours infiltrate the caudal vena cava (CVC), renal vein or phrenicoabdominal vein, with the intravascular infiltration rate of PHEO and adrenocortical tumours being 14−88% (Massari et al., 2011, Lang et al., 2011, Pey et al., 2022, Yoshida et al., 2016, Nagumo et al., 2020, Davis et al., 2012, Barrera et al., 2013, Kyles et al., 2003, Herrera et al., 2008, Mayhew et al., 2019, Knight et al., 2019) and 2−29% (Massari et al., 2011, Lang et al., 2011, Pey et al., 2022, Yoshida et al., 2016, Nagumo et al., 2020, Davis et al., 2012, Barrera et al., 2013, Kyles et al., 2003, Mayhew et al., 2019, Knight et al., 2019), respectively. Budd−Chiari‐like syndrome (BCLS) is characterized by portal hypertension, hepatomegaly and ascites resulting from mechanical obstruction or stenosis of the hepatic vein, CVC outflow tract and right atrium (Grooters & Smeak, 1995). It is associated with canine PHEO involving vascular invasion into the CVC (Schwartz et al., 2008, Schoeman & Stidworthy, 2001, Rosa et al., 2012).

Although there are a few reports on radiation therapy for large adrenal tumours with intravascular invasion (Dolera et al., 2016, Maruo et al., 2019), surgery is considered to be the definitive treatment for canine adrenal PHEO (Lunn & Boston, 2020). Two case reports have described en bloc resection, including the CVC, for canine PHEO infiltrating the CVC (Louvet et al., 2005, Guillaumot et al., 2012). To our knowledge, there have been no reports regarding en bloc resection of such a PHEO, including the surrounding tissues, based on a preoperative surgical plan using computed tomography (CT) findings. Therefore, the purpose of this report was to describe a dog with PHEO which had BCLS and underwent en bloc tumour resection based on a preoperative surgical plan following the CT, including the right hepatic division, caval thrombus and segmental CVC, with good outcome.

## 2 MATERIALS AND METHODS

A 13‐year‐old castrated male miniature dachshund was brought to an emergency animal clinic with a chief complaint of abdominal distension and appetite loss. An abdominal ultrasound revealed a large mass in the abdominal cavity. The patient was examined at another animal hospital and underwent CT and fine‐needle aspiration of the mass, which was suspected to be a malignant epithelial tumour that originated from the right adrenal gland and had severely infiltrated into the surrounding tissues. Approximately 2 weeks later, the patient was referred to our hospital for surgical treatment.

On the first evaluation, the patient had anorexia, asthenia and lethargy, and a general physical examination revealed severe abdominal distension and a body condition score of 2/5. The patient had a body weight of 9.5 kg, a rectal temperature of 38.1°C, a heart rate of 124 bpm and a respiratory rate of 28 bpm. Haematology and serum chemistry profiles are shown in Table 1. A complete blood count (CBC) revealed a non‐regenerative anaemia with a packed cell volume (PCV) of 20%. Serum chemistry showed decreased total protein (4.9 g/dL) and increased alanine aminotransferase level (163 U/L), potassium level (5.2 mEq/L) and ammonia level (119 μg/dL). Blood coagulation testing revealed increased D‐dimer levels (25.85 μg/mL). Urine testing showed no obvious abnormalities, including a specific gravity over 1.040. Abdominal radiography revealed that the gastrointestinal tract was caudally displaced by a large intra‐abdominal mass; there was poor visceral peritoneal detail and uniform ground‐glass opacities in the abdominal cavity. Chest radiography indicated no lung nodules suggestive of metastases. Abdominal ultrasound revealed severe ascites and suspected partial liver infiltration by the intra‐abdominal mass, with the abundant vascular flow. The patient was subsequently admitted to our hospital for supportive care including a blood transfusion.

**TABLE 1:** Haematology and serum chemistry profiles

Parameter		Unit	Value	Normal range
RBC		10^6^/μL	3.5	5.5–8.5
PCV		%	20	37–55
WBC		/μL	12,600	6000–17,000
	Stab	/μL	378	0–300
	Seg	/μL	9954	3000–11,500
	Lym	/μL	567	1000–4800
	Mono	/μL	1575	150–1350
	Eos	/μL	126	100–750
Plt		10^3^/μL	401	200–500
TP		g/dL	4.9	5.2–8.2
Alb		g/dL	2.6	2.7–3.8
Glu		mg/dL	94	77–125
AST		U/L	24	0–50
ALT		U/L	163	10–100
ALP		U/L	142	23–212
GGT		U/L	5	0 – 7
BUN		mg/dL	14.6	7–27
Cr		mg/dL	0.57	0.5–1.8
NH_3_		μg/dL	119	16–75
Na		mEq/L	143	134–153
K		mEq/L	5.2	3.4–4.6
Cl		mEq/L	107	105–118
Ca		mg/dL	8.9	9.3–12.1
P		mg/dL	5.0	1.9–5.0
CRP		mg/dL	0.75	0–1.0
APTT		s	13.9	10–16
PT		s	5.6	4 – 6
Fib		mg/dL	177.8	86–375
D‐dimer		μg/mL	25.85	0–2.0
ATIII		%	145	102–156

Abbreviations: Alb, albumin; ALP, alkaline phosphatase; ALT, alanine aminotransferase; APTT, activated partial thromboplastin time; AST, aspartate aminotransferase; ATIII, antithrombinIII; BUN, blood urea nitrogen; Cr, creatinine; CRP, c‐reactive protein; Eos, eosinophil; Fib, fibrinogen; GGT, gamma‐glutamyltransferase; Lym, lymphocyte; Mono, monophil; NH3, ammonia; PCV, packed cell volume; Plt, platelet count; PT, prothrombin time; RBC, red blood cell count; Seg, segmented neutrophil; Stab, stab neutrophil; TP, total protein; WBC, white blood cell count.

The day after the first evaluation, the anaemia (PCV 20%) noted on the initial CBC improved after 200 mL fresh whole blood was transfused to a PCV of 29%, and the patient showed increased activity. Additionally, 1012 mL of peritoneal fluid was removed by abdominal paracentesis under ultrasound guidance. The peritoneal fluid analysis revealed the following findings: PCV, 0.2%; total protein, 4.0 g/dL; albumin level, 1.9 g/dL; cell count, 600 /μL; and specific gravity, 1.030. There were no neoplastic cells in the sediment smears. An adrenal hormone test was performed as shown in Table 2. The adrenocorticotropic hormone (ACTH) stimulation test was within the normal ranges. In addition, plasma catecholamine fraction, including adrenaline, noradrenaline and dopamine, was not high.

**TABLE 2:** Adrenal hormone test

ACTH stimulation test		Unit	Value
Cortisol	pre	μg/dL	0.7
	post	μg/dL	8.0

Plasma catecholamine fraction			
Adrenaline		pg/mL	106
Noradrenaline		pg/mL	217
Dopamine		pg/mL	17

During hospitalization, medical treatment was continued using carbazochrome sodium sulfonate (ADONA^®^; Nipro Co, Osaka, Japan), menatetrenone (Kaytwo^®^N; Eisai Co, Tokyo, Japan) and famotidine (Gaster^®^; LTL Pharma Co, Tokyo, Japan). The patient's physical condition did not improve, and re‐accumulation of ascites was noted. Therefore, we proposed surgical treatment, and the owner then provided consent for en bloc resection of the mass. Five days after the first evaluation, the patient underwent a CT examination, followed by surgery.

Under general anaesthesia, the patient firstly underwent contrast‐enhanced CT. A 24 G indwelling needle (Surflow^®^; Terumo Co, Tokyo, Japan) was placed in the cephalic vein of the patient's left and right anterior limbs. The following pre‐anaesthesia medications were subcutaneously injected: 0.04 mg/kg of atropine sulphate (Mitsubishi Tanabe Pharmaceutical Co, Osaka, Japan), 1.0 mg/kg of maropitant (Zoetis, Parsippany, NJ, USA), 1.0 mg/kg of famotidine and 1.0 mg/kg of prednisolone (Kyoritsu Seiyaku Co, Tokyo, Japan). Anaesthesia induction was brought by the intravenous administration of 5.0 μg/kg of fentanyl citrate (Terumo Co) followed by 4.7 mg/kg of propofol (Mylan; Mylan Pharmaceutical Co, Tokyo, Japan). Tracheal tube intubation was performed for mechanical ventilation with 0.3−1.1% isoflurane (IsoFlo; Zoetis) and pure oxygen (2 L/min).

**FIGURE 1:**
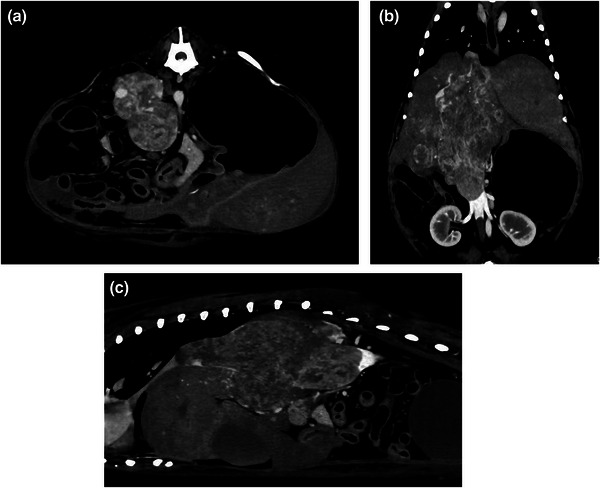
Preoperative computed tomography images. (a) Cross‐sectional image. (b) Coronal image. (c) Sagittal image. The tumour arising from the right adrenal gland not only invades the surrounding tissue but also forms a large caval thrombus in the caudal vena cava, spreading into the right hepatic vein and accessory right hepatic vein. The tip of the caval thrombus reaches the diaphragm; moreover, there is poor perfusion of the left and central hepatic veins and reduced hepatic blood perfusion, which resulted in a poor contrast‐enhancement.

**FIGURE 2:**
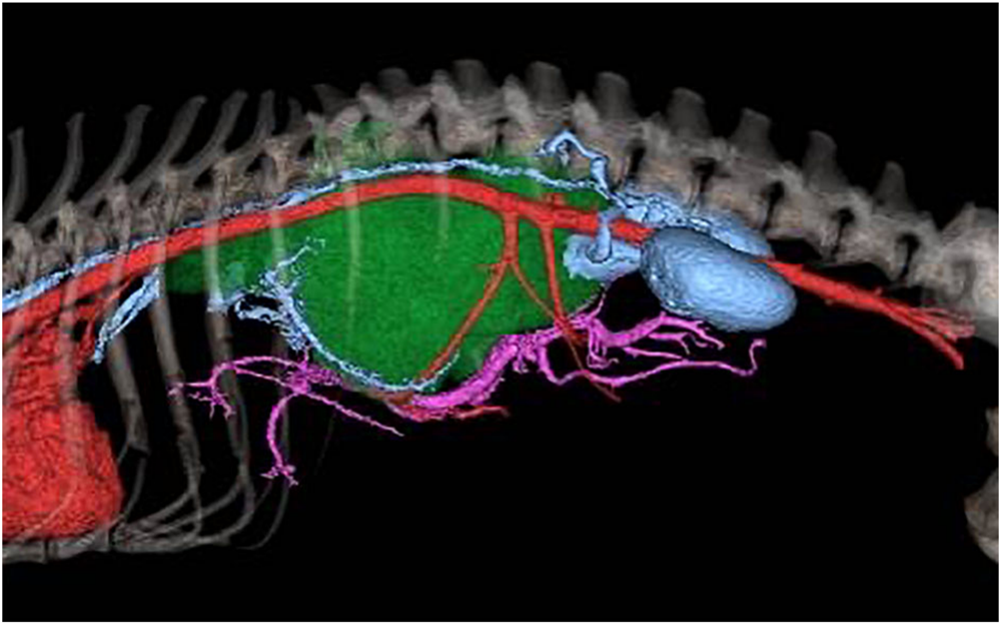
Preoperative three‐dimensional computed tomography image. A large tumour arising from the right adrenal gland fills the caudal vena cava. It was presumed that the interruption of the blood flow caused the formation of a collateral vessel to the azygos vein.

Contrast‐enhanced CT revealed irregular margins of the right adrenal tumour, caval thrombus formation in the CVC with the right adrenal tumour as the primary lesion and infiltration from the hepatic vein into the right hepatic division (Figure 1). There was overall liver enlargement with poor contrast enhancement; the right hepatic vein, accessory right hepatic vein and accessory central hepatic vein were not visible. The caval thrombus filled in the CVC and showed invasion from the cranial side of the confluence of the right renal vein beyond the diaphragm. Furthermore, some collateral circulation from the CVC to the azygos vein naturally occurred near and caudal to the confluence of the renal veins (Figure 2). In addition, the caval thrombus partially occluded the left and central hepatic veins and impaired blood flow in the hepatic vein. There was an enlargement of sternal lymph nodes; however, there was no evidence of pulmonary metastasis.

**FIGURE 3:**
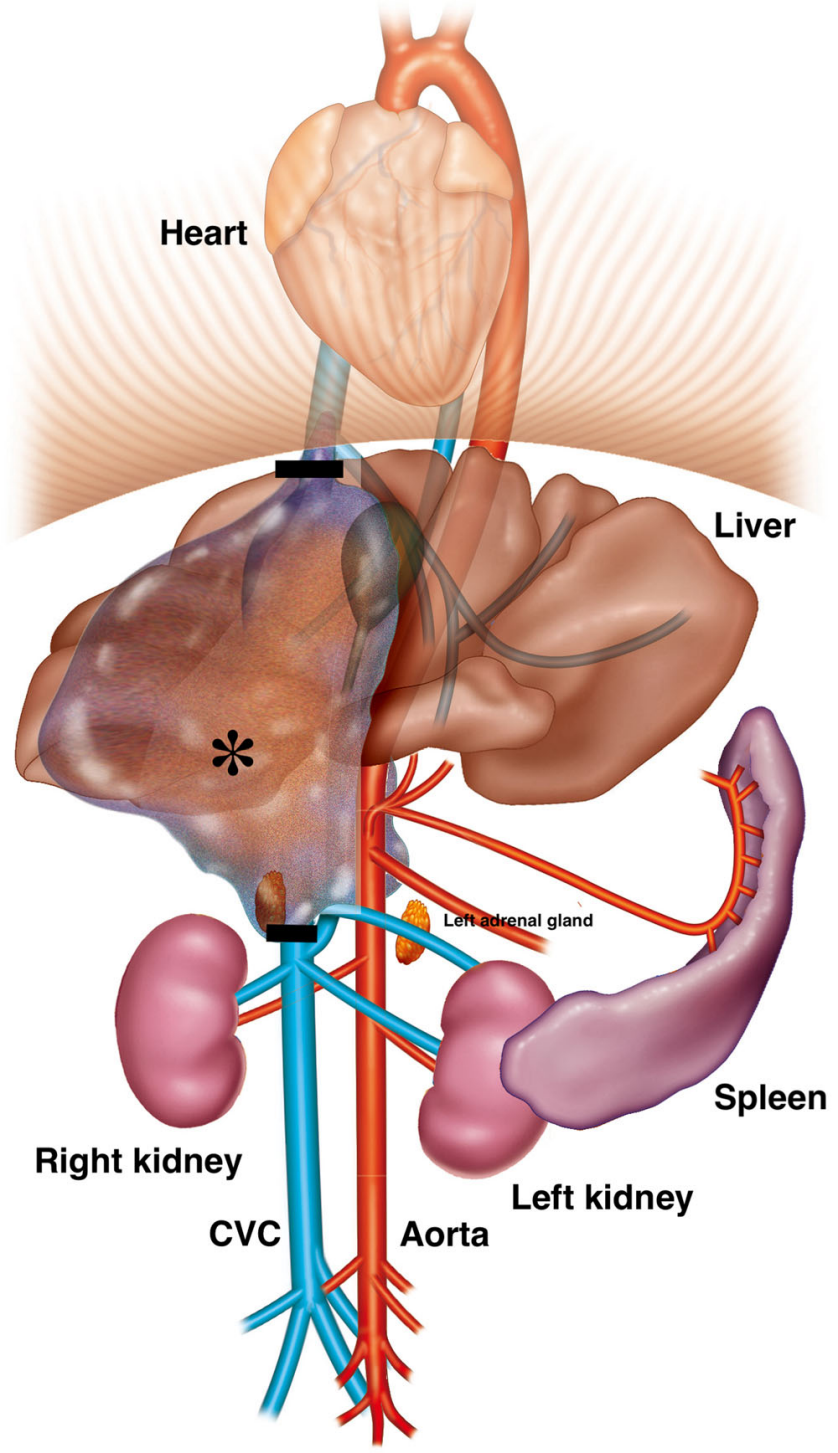
Illustration of the right adrenal tumour. Computed tomography images allowed us to determine the extent of right adrenal tumour and, therefore, facilitated the surgical planning. Two black lines indicate the cutting sites of caudal vena cava (CVC). We expected that en bloc resection of the right adrenal mass involving the right hepatic division, caval thrombus and segmental CVC would be feasible due to the azygos bypass of the caudal venous return.

Figure 3 illustrates the extent of the right adrenal tumour. A surgical plan was formulated based on the CT findings as follows: en bloc resection of the right adrenal mass involving the right hepatic division, with caval thrombus and segmental CVC being feasible due to the azygos bypass of the caudal venous return. To achieve this, a surgical incision at the two sites of CVC between the thrombus and confluence of the right renal vein and between the central and right hepatic divisions would be performed. Informed owner consent for the surgical plan formulated based on the CT findings was obtained. The patient was, therefore, moved to the surgical preparation room maintaining general anaesthesia, and aseptic surgery was prepared.

A 6‐Fr catheter (Atom multipurpose tube; Atom Medical Co, Tokyo, Japan) was placed in the femoral artery and vein for blood pressure measurement. The patient was then moved to the operating room and positioned in dorsal recumbency on the operating table. A small skin incision was made in the right femoral trigon for isolating the femoral artery and vein. A 6‐Fr catheter (Atom multipurpose tube; Atom Medical Co) was placed in the femoral artery and vein for the measurement of arterial and venous pressure, respectively. During surgery, blood pressure was monitored by placing catheters in the right jugular vein and femoral artery and vein. For hemodynamic stability during surgery, dopamine hydrochloride (Teva Takeda Pharma Co, Nagoya, Japan), dobutamine hydrochloride (Kyowa Pharmaceutical Industry Co, Osaka, Japan), carperitide (atrial natriuretic peptide; Daiichi‐Sankyo Co, Tokyo, Japan), phentolamine mesilate (Regitin^®^; Novartis Pharma AG, Basel, Switzerland), phenylephrine hydrochloride (Neo‐Synesin; Kowa Co, Tokyo, Japan) and noradrenaline (Alfresa Pharma Co, Osaka, Japan) were used. Fresh whole blood transfusion and continuous rate infusion (CRI) of crystalloid solution (Solita^®^‐T1; Awai Pharma Co, Tokyo, Japan) 1−10 mL/kg/h were performed. Intraoperative and postoperative analgesia management was performed using remifentanil hydrochloride (Maruishi Pharmaceutical Co) 5−30 μg/kg/h and fentanyl citrate 1.25−2.5 μg/kg/h, respectively. In addition, CRIs of nafamostat mesilate (serine protease inhibitor; Awai Pharma Co) 0.2 mg/kg/h and lidocaine hydrochloride (Aspen Japan Co, Tokyo, Japan) 25−50 μg/kg/min were performed. Ampicillin (Meiji Seika Pharma Co, Tokyo, Japan) was intravenously administered at the dose of 20 mg/kg during anaesthesia induction and at 2‐h intervals.

**FIGURE 4:**
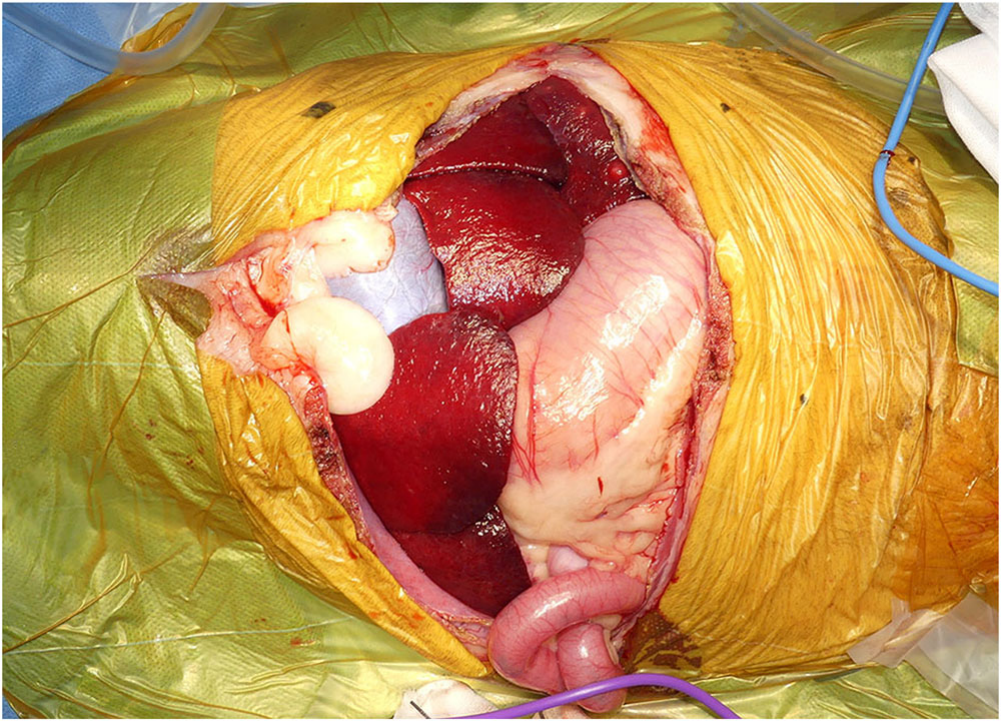
Abdominal Mercedes incision (cranial midline celiotomy and bilateral paracostal incisions) in the patient.

**FIGURE 5:**
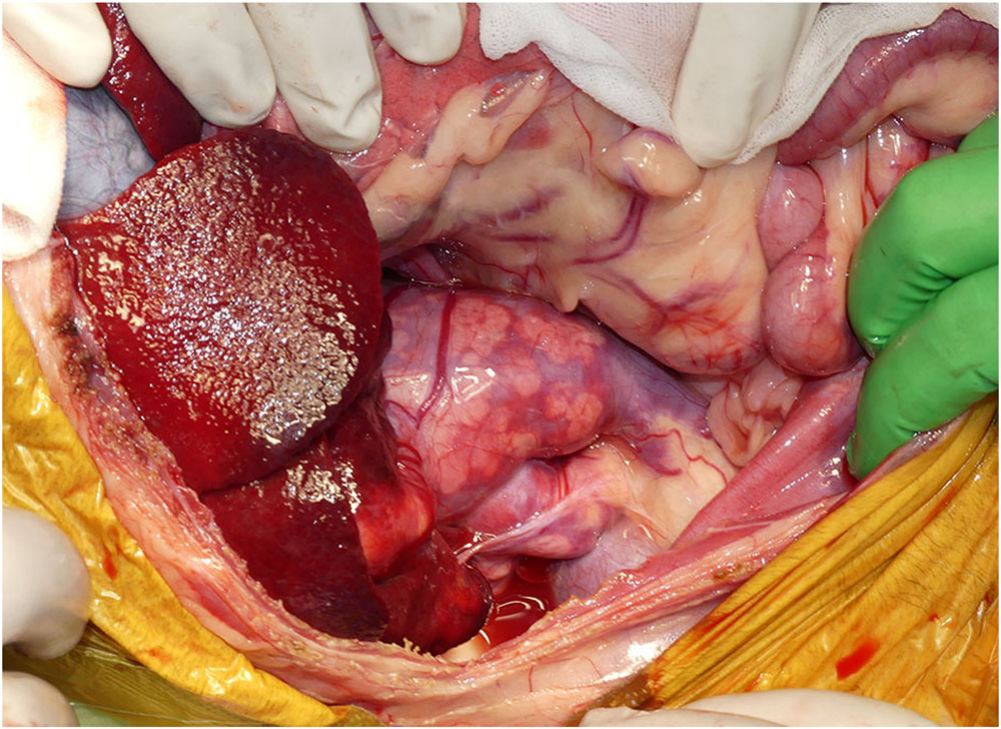
Intraoperative findings. A large right adrenal tumour integrated with the caudate lobe and a large caval thrombus are observed.

**FIGURE 6:**
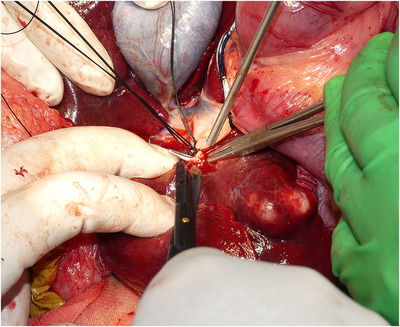
The blood vessels and hepatic ducts that flow into the right hepatic division were ligated and resected en bloc.

**FIGURE 7:**
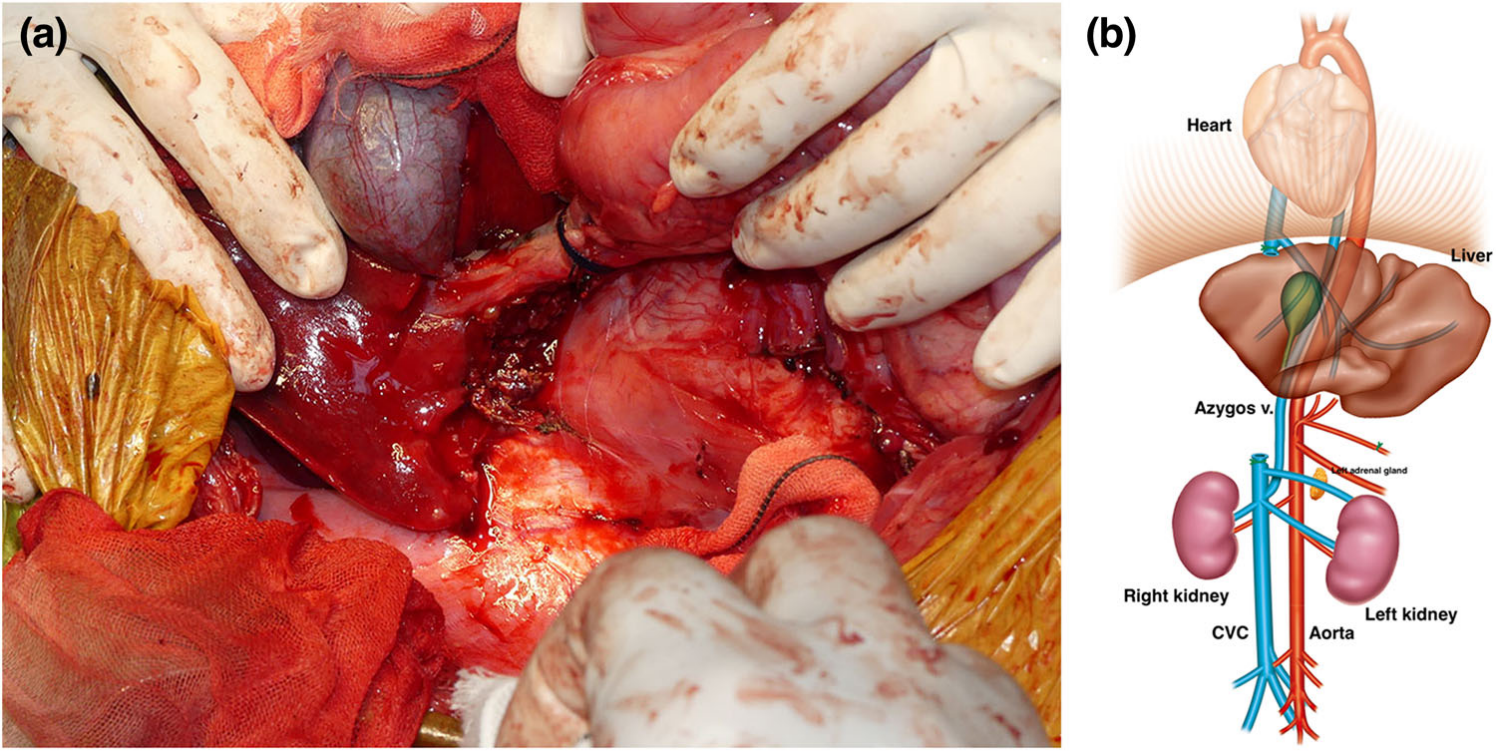
Intraoperative findings and illustrations after removal. (a) Intraoperative findings. (b) Illustration. The right adrenal tumour was resected en bloc with the right hepatic division, caudal vena cava and caval thrombus.

A Mercedes incision (combination of cranial celiotomy and paracostal incisions), a caudal median sternotomy and a diaphragm midline incision were performed to approach the mass (Figure 4). The gross findings revealed a large mass originating from the right adrenal gland with abundant neovascularization and a caval thrombus infiltrating the CVC (Figure 5). The hepatorenal ligament, right triangular ligament and falciform ligament of the liver were dissected. While caudally retracting the right kidney using a malleable retractor, the mass was isolated using monopolar and bipolar electrocauteries and a vessel sealing system (Force Triad^TM^; Medtronic, Minneapolis, MN, USA). Using umbilical tape, a tourniquet was placed around Glisson's sheath, including the hepatic arteries, portal vein and common bile duct in the hilar region for Pringle manoeuvre (tourniquet 1). Two other tourniquets were placed, one around the thoracic CVC (tourniquet 2) and another around the CVC in the cranial site of the confluence of the right renal vein (tourniquet 3). The femoral vein pressure was 7 mmHg, and then, decreased to 4 mmHg after tourniquet 3 was temporarily engaged. Tourniquet 3 was released, and the Pringle manoeuvre was performed by squeezing tourniquet 1. The hepatic arteries, portal branch and hepatic duct in the right hepatic division were *en masse* ligated and resected using 3‐0 coated braided nylon sutures (Surgilon^TM^; Medtronic), and then, tourniquet 1 was released to discontinue the Pringle manoeuvre (Figure 6). Furthermore, the mass was isolated from the surrounding tissues. Tourniquet 3 was engaged to expose the caval thrombus through a T‐shaped incision in the CVC. Subsequently, the CVC between caval thrombus and tourniquet 3 was resected through a circumferential incision, and then, the caudal side of CVC was completely closed with a transfixation ligature using 3‐0 polydioxanone sutures (PDS II^®^; Johnson & Johnson, New Brunswick, NJ, USA). Tourniquets 1 and 2 were engaged for a second Pringle manoeuvre and blockage of the thoracic CVC, followed by the resection of the CVC between the central and right hepatic divisions. After traction and removal of the caval thrombus from the CVC, the caudate papillary process was dissected using an ultrasonically activated scalpel (Sonosurg; Medical Space Inc, Koshigaya, Japan) and Force Triad^TM^ (Medtronic), and small vessels from the caudate papillary process were ligated using 5‐0 polypropylene suture (PROLENE^®^; Johnson & Johnson), to achieve en bloc resection of the mass, including the right hepatic division, caval thrombus and segmental CVC (Figure 7). Immediately after this, the cranial resection stump of the CVC was completely closed with a simple continuous suture pattern using 5‐0 polypropylene suture (PROLENE^®^; Johnson & Johnson), followed by the release of tourniquets 1 and 2.

Since several white nodules were found in the spleen, splenectomy was subsequently performed using Force Triad^TM^ (Medtronic). A chest tube was then placed through the abdominal wall and diaphragm into the thoracic cavity. The incised diaphragm was sutured in a continuous pattern using 2‐0 absorbable sutures (PDS II^®^; Johnson & Johnson). The median sternotomy was closed with an interrupted cruciate pattern using 0 absorbable sutures (PDS II^®^; Johnson & Johnson) and the abdominal wall, subcutaneous tissues and skin were routinely closed.

## 3 RESULTS

The en bloc resection of the right adrenal mass involving the right hepatic division, caval thrombus and segmental CVC was feasible in this case. The operation time from skin incision to closure was 162 min. The first and second Pringle manoeuvres, used for vascular blockage to reduce haemorrhage, lasted 5 min 20 s and 16 min 56 s, respectively, while the thoracic CVC blockage time was 17 min 27 s. There was no tachycardia during en bloc resection.

During anaesthesia, up to 7.5 μg/kg/min of dopamine and dobutamine and 0.05−0.2 μg/kg/min of carperitide were firstly infused for the stabilization of the patient's hemodynamics. After placing the tourniquet on the CVC, the mean arterial blood pressure increased from 61 to 85 mmHg. Accordingly, 0.02 mg/kg of phentolamine was administered intravenously, with the mean arterial blood pressure decreasing to 71 mmHg. Immediately after performing the first Pringle manoeuvre, the mean arterial blood pressure increased to 91 mmHg. Therefore, 0.02 mg/kg of phentolamine was repeated intravenously, with the mean arterial blood pressure decreasing to 72 mmHg. After the caudal tourniquet of the CVC blockade, following the release of the Pringle manoeuvre, the mean arterial blood pressure further decreased to 48 mmHg. A 5 μg/kg dose of phenylephrine was administered intravenously as needed. During surgery, blood transfusion was performed through intravenous drip infusion at 1−5 mL/kg/h due to persistent bleeding; a bolus was administered when bleeding increased. The total intraoperative blood transfusion volume reached 350 mL. During the second Pringle manoeuvre and thoracic CVC blockade, the mean arterial blood pressure decreased to 39 mmHg; 5−20 μg/kg phenylephrine was administered. Since the mean arterial blood pressure was as low as 49 mmHg after releasing all vascular blockages, 20−50 μg/kg phenylephrine was administered again. Additionally, noradrenaline was continuously infused at the dose of 0.125−0.5 μg/kg/min.

The patient recovered well from anaesthesia, with a mean arterial blood pressure of 71 mmHg and a sinus cardiac rhythm, without specific postoperative problems. The blood transfusion was continued postoperatively, with an overall administration of 550 mL during the perioperative period. The patient could stand up the day after the operation and showed a good response to surgery.

**FIGURE 8:**
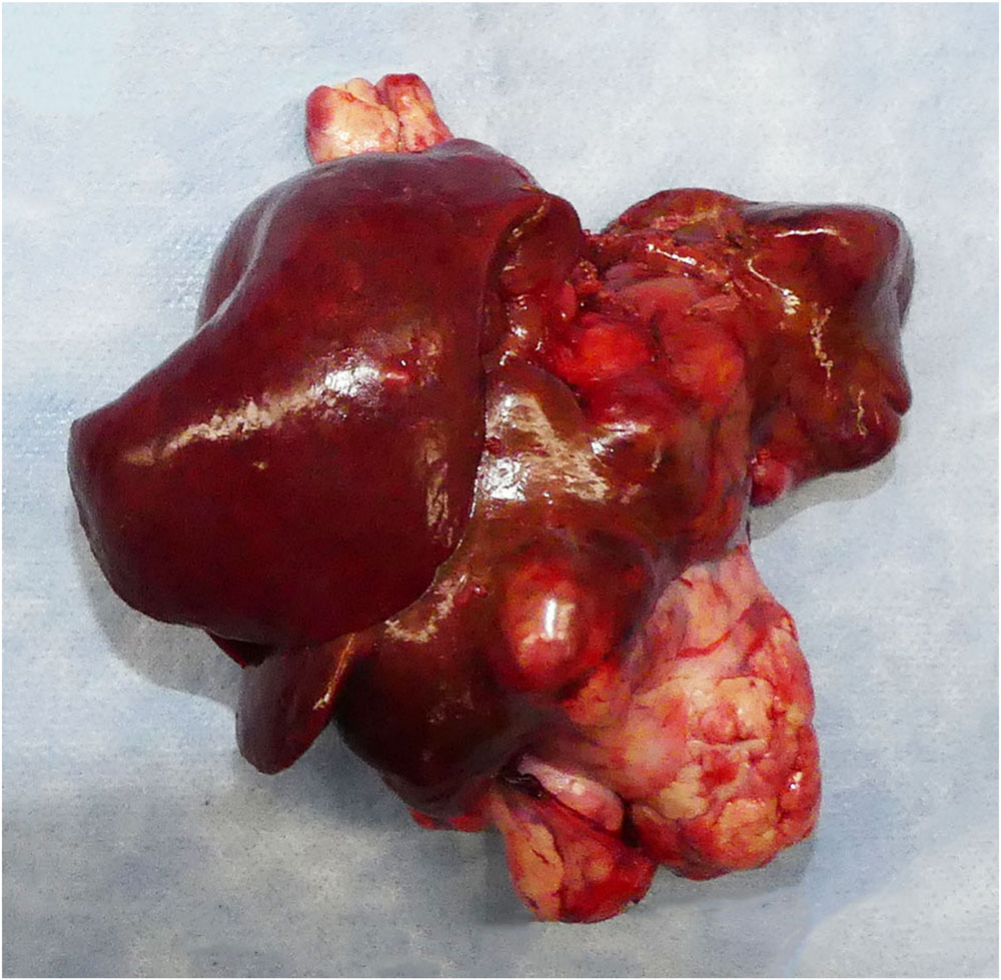
The resected tumour.

The size of the excised tissue was 12.3 cm × 12.8 cm × 8.4 cm (total weight: 341 g), and the tumour was completely resected grossly (Figure 8). Histopathologic examination revealed a large PHEO with an indistinct border and invasion into the liver parenchyma and blood vessels. There were no tumour cells in the hepatic surgical margin, but metastasis was found in the splenic capsule. Mitotic count within the tumour was 2/10 high‐power fields.

The preoperative abdominal distension resolved after surgery. There was a postoperative improvement in both appetite and body weight. The patient's general condition was good during postoperative hospitalization, with an ACTH stimulation test on a postoperative day (POD) 3 yielding values within the normal ranges (pre: 0.7 μg/dL; post 1 h: 8.0 μg/dL). On POD 4, the CBC showed a PCV of 37%, indicating the improvement of anaemia, and the chest tube was removed. On POD 10, thyroid hormones were measured and found to be low (T4: < 0.3 μg/dL; FT4: < 0.3 pg/dL; TSH: 1.3 ng/mL). The patient was discharged on POD 12 and was prescribed famotidine, maropitant, levothyroxine (Aska Pharmaceutical Co, Tokyo, Japan) and faropenem (Maruho co, Osaka, Japan). On POD 19, the patient's clinical signs, including appetite, fully improved, and all prescriptions other than levothyroxine were discontinued.

On POD 68, the patient visited our hospital with a chief complaint of ataxia. Contrast‐enhanced CT images suggested metastases to the sternal lymph nodes, lung, left/central liver divisions, hepatic lymph nodes and C3–C5 spine regions. On POD 130, the patient died due to suspected metastases and cachexia.

## 4 DISCUSSION

Herein, a PHEO invaded the CVC intravascularly to form a caval thrombus. Preoperative contrast‐enhanced CT revealed an intrahepatic contrast‐enhancement effect, which suggested hepatic vein blockage by a caval thrombus. Given the collateral circulation to the azygos vein, the caval thrombus was considered to be severely obstructive to blood flow. Furthermore, BCLS could have resulted from the accumulation of modified transudative ascites and hepatomegaly caused by mechanical obstruction of the hepatic vein and CVC due to the PHEO infiltrating the CVC.

The superior effectiveness of CT in the detection of intravascular infiltrating lesions in canine adrenal tumours has been shown to be as follows: ultrasound exam has a detection sensitivity of 80−100% and specificity of 90−96% (Davis et al., 2012, Kyles et al., 2003), while CT has a detection sensitivity of 92% and specificity of 100% (Schultz et al., 2009). CT may effectively detect collateral circulation due to CVC obstruction. A previous study on healthy dogs reported that 3 weeks after gradual obstruction of the CVC in the cranial region of the renal vein, contrast CT revealed collateral circulation from the renal capsule and the CVC to the lumbar, vertebral and azygos veins (Peacock et al., 2003). In our case, since the collateral circulation returned from the CVC to the azygos vein was created, we considered that the venous return volume could be maintained even with complete CVC obstruction. Based on the CT findings, we could plan an en bloc resection of the right hepatic division, caval thrombus and right adrenal tumour, including a part of the CVC, without the reduction of venous return.

The presence or absence of caval thrombi in canine adrenal tumours is considered a prognostic factor (Massari et al., 2011). PHEO is one of the short‐term prognostic factors since intravascular invasion into the CVC is common (Barrera et al., 2013). On the other hand, caval thrombus removal through CVC incision in canine adrenal tumours is not associated with short‐term prognosis (Schwartz et al., 2008, Kyles et al., 2003, Herrera et al., 2008). In dogs with caval thrombi, those with intravascular infiltration beyond the hilar region are at least four times more likely to die within a short period than those without (Barrera et al., 2013). It has been recently demonstrated that even dogs with an intravascularly infiltrated caval thrombus beyond the hepatic hilum can survive the perioperative period secondary to skilled anaesthesia management by anaesthesiologists and blood preparations (Lipscomb, 2019). In dogs with adrenal tumours undergoing surgical treatment, the perioperative complication and mortality rates are ≥ 50% (Barthez et al., 1997, Kyles et al., 2003, Mayhew et al., 2019, Knight et al., 2019) and 12−50% (Massari et al., 2011, Lang et al., 2011, Schwartz et al., 2008, Barrera et al., 2013, Kyles et al., 2003, Herrera et al., 2008, Mayhew et al., 2019, Knight et al., 2019, Lang et al., 2011), respectively. In our case, contrast‐enhanced CT suggested poor systemic blood circulation due to caval thrombus. In addition, since our patient also had BCLS, the patient had the potential risk of higher perioperative mortality. When an experienced surgeon performs the appropriate procedure, the presence or absence of a caval thrombus does not significantly affect the perioperative complication/mortality rate (Kyles et al., 2003). Previous reports of en bloc resection, including the CVC for PHEO with intravascular infiltration into the CVC, have reported a good long‐term prognosis, with a survival time of 20−49 months (Louvet et al., 2005, Guillaumot et al., 2012). On the other hand, other previous cases of canine PHEO with BCLS have suggested a poor prognosis: one patient died during the perioperative period due to dyspnoea (Kyles et al., 2003), while another underwent euthanasia at the owner's request (Schoeman & Stidworthy, 2001, Rosa et al., 2012). Our case with preoperative BCLS also showed a short survival time (approximately 4 months) after surgery, and the causes of death were suspected to be metastases and cachexia. In our case, BCLS postoperatively disappeared because the resumption of flow through the left and central hepatic divisions likely led to the resolution of the ascites. In addition, the surgical treatment improved the appetite loss and overall condition of the patient for a short postoperative time. Therefore, en bloc resection of PHEO with caval thrombus causing BCLS should only be considered to improve the quality of life in patients with collateral venous return development.

In conclusion, our report described that preemptively planned en bloc resection of an extensive right adrenal PHEO, involving the right hepatic division, caval thrombus and segmental CVC, was successfully performed based on the preoperative CT findings in the patient, and BCLS resolved after surgery. In the case of CT findings indicating naturally occurring collateral vessels, en bloc resection could be feasible for PHEO patients with extensive infiltration into the CVC, right hepatic division and hepatic vein obstruction.

## AUTHOR CONTRIBUTIONS

Ryo Takeuchi: conceptualization (supporting); data curation (supporting); investigation (supporting); methodology (supporting); visualization (lead); writing—original draft preparation (equal); writing—review & editing (equal). Kumiko Ishigaki: conceptualization (supporting); data curation (supporting); investigation (supporting); methodology (supporting); project administration (supporting); supervision (supporting); visualization (supporting); writing—original draft preparation (equal); writing—review & editing (equal). Orie Yoshida: conceptualization (supporting); data curation (supporting); investigation (supporting); methodology (supporting); supervision (supporting); visualization (supporting); writing—original draft preparation (equal); writing—review & editing (equal). Naoki Sakurai: conceptualization (supporting); data curation (supporting); investigation (supporting); methodology (supporting); visualization (supporting); writing—original draft preparation (equal); writing—review & editing (equal). Kazuyuki Terai: conceptualization (supporting); data curation (supporting); investigation (supporting); methodology (supporting); visualization (supporting); writing—original draft preparation (equal); writing—review & editing (equal). Tatsuya Heishima: conceptualization (supporting); data curation (supporting); investigation (supporting); methodology (supporting); visualization (supporting); writing—original draft preparation (equal); writing—review & editing (equal). Kazushi Asano: conceptualization (lead); data curation (lead); investigation (lead); methodology (lead); project administration (lead); supervision (lead); visualization (supporting); writing—original draft preparation (equal); writing—review & editing (equal).

## CONFLICT OF INTEREST STATEMENT

The authors declare no conflicts of interest.

## FUNDING INFORMATION

No third‐party funding or support was received in connection with this study or the writing or publication of the manuscript.

### ETHICS APPROVAL STATEMENT

Informed owner consent of the patient was obtained prior to the first evaluation, and all procedures for the patient were approved by the Ethical Committee of Nihon University Animal Medical Center (accession No. 03‐019).

### PEER REVIEW

The peer review history for this article is available at https://publons.com/publon/10.1002/vms3.1110.

## Data Availability

All data supporting the conclusions of this article are included within the article.
